# Applying the Host-Microbe Damage Response Framework to *Candida* Pathogenesis: Current and Prospective Strategies to Reduce Damage

**DOI:** 10.3390/jof6010035

**Published:** 2020-03-11

**Authors:** Paul L. Fidel, Junko Yano, Shannon K. Esher, Mairi C. Noverr

**Affiliations:** 1 Center of Excellence in Oral and Craniofacial Biology, Louisiana State University Health Sciences Center School of Dentistry, New Orleans, LA 70119, USA; JYano@lsuhsc.edu; 2 Department of Microbiology and Immunology, Tulane University School of Medicine, New Orleans, LA 70112, USA; sesher1@tulane.edu (S.K.E.); mnoverr@tulane.edu (M.C.N.)

**Keywords:** *Candida albicans*, pathogenesis, host, immune response, damage response

## Abstract

Disease is a complex outcome that can occur as a result of pathogen-mediated damage, host-mediated damage or both. This has led to the revolutionary concept of the damage response framework (DRF) that defines microbial virulence as a function of host immunity. The DRF outlines six scenarios (classes) of host damage or beneficial outcomes, depending on the microbe and the strength of the immune response. *Candida albicans* is uniquely adapted to its human host and can exist as either a commensal, colonizing various anatomical sites without causing notable damage, or as a pathogen, with the ability to cause a diverse array of diseases, ranging from mucosal to invasive systemic infections that result in varying levels of microbe-mediated and/or host-mediated damage. We recently categorized six different forms of candidiasis (oropharyngeal, hematogenous, intra-abdominal, gastrointestinal, denture stomatitis, and vulvovaginitis) into independent DRF classes, supporting a contemporary view of unique mechanisms of pathogenesis for these *Candida* infections. In this review, we summarize the evidence for the pathogenesis of these various forms of candidiasis in the context of the DRF with the further intent to provide insights into strategies to achieve a level of host response or outcome otherwise, that limits host damage.

## 1. Introduction

**Figure 1:**
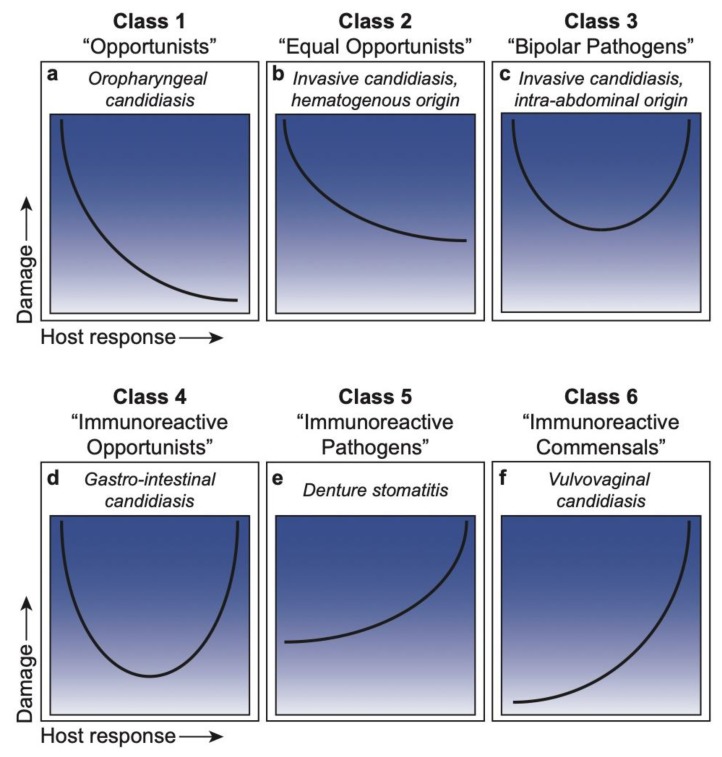
Damage-response framework curves for each class and associated *Candida* infections. (**a**) Class 1: ‘Opportunists’, or pathogens that cause damage only in the setting of weak host responses; represented by oropharyngeal candidiasis. (**b**) Class 2: ‘Equal Opportunists’, or pathogens that cause damage in hosts with weakened immune responses or in the setting or normal responses; represented by invasive candidiasis of hematogenous origin. (**c**) Class 3: ‘Bipolar Pathogens’, or pathogens that cause damage under appropriate immune responses, which is amplified at both ends of the immune spectrum; represented by invasive candidiasis of intra-abdominal origin. (**d**) Class 4: ‘Immunoreactive Opportunists’, or pathogens that cause damage primarily in the extremes of weak and strong host responses; represented by gastro-intestinal candidiasis. (**e**) Class 5: ‘Immunoreactive Pathogens’, or pathogens that cause damage across the spectrum of immune responses, with enhanced damage under strong host responses; represented by denture stomatitis. (**f**) Class 6: ‘Immunoreactive Commensals’, or pathogens that cause damage only under strong host responses; represented by vulvovaginal candidiasis.

Historically, the nature and extent of host damage by an opportunistic microbe were considered highly dependent on virulence attributes of the microbe. However, it is now quite clear that damage to the host during infection is also reflective of the immune status of the host and often contributed to by host responses. Therefore, disease itself is a complex outcome, which can arise because of pathogen-mediated damage, host-mediated damage or both. Hence, in many interactions between pathogens and normal hosts, there is a continuum between pathogen-mediated and host-mediated damage, which results in disease only when the nature of the damage impairs the normal function of the host. This has led to the concept of the damage response framework (DRF) [1], which defines microbial pathogenesis as a function of the outcome of an interaction between a host and a microbe. The DRF is based on the core principle that there are no exclusive pathogens, commensals, or opportunists, but that microbial pathogenesis requires a microbe and a host to interact, with the relevant outcome being damage to the host. In a global context, the DRF shifted the focus away from exclusively microbe-mediated damage and emphasized the role of the host as a contributor to damage. Importantly, in addition to the host and microbe, this flexible conceptual framework also includes outcomes that are a function of multiple variables, such as environment and time. Together, the DRF categorizes microbial pathogens into six classes, based on six distinct damage response curves, which portray host damage as a function of the host response (Figure 1). Further, host-mediated damage is contributed to by weak or strong immune responses to the microbes. 

*Candida albicans* most often exists as a normal member of the microbiota at various mucosal sites [2,3]. When host and environmental conditions permit growth and/or translocation of *C. albicans,* pathogenesis ensues and causes host damage, leading to a wide range of diseases, both mucosal and systemic, in either immunocompetent or immunocompromised individuals [4,5]. Each type of disease has a unique signature with outcomes associated with varying levels of host response that result in varying damage or potential benefit to the host. Accordingly, the versatility of *C. albicans*, with its commensal presence and pathogenic potential at various anatomical sites, positions it in a unique situation to fit within each of the DRF classes [1,6] that collectively define *Candida* pathogenesis. This includes oropharyngeal candidiasis in DRF Class 1, systemic candidiasis of hematogenous origin in Class 2, systemic candidiasis of intra-abdominal origin in Class 3, gastrointestinal candidiasis in Class 4, denture stomatitis in Class 5, and vulvovaginal candidiasis in Class 6 (Figure 1).

In this review, we again revisit the DRF concept in the context of these anatomically distinct *Candida* infections [7]. As we describe *Candida* infections reflecting each of the six classes of the DRF, we also provide current and/or prospective strategies to achieve a level of host response that reduces host damage. Key terms/abbreviations used throughout the review are defined in backmatter. 

## 2. The Damage Response Framework

### DRF Classification of Microbial Species

Class 1: “Pathogens that only cause damage in the setting of weak immune responses”. Microorganisms placed in this class are those usually considered opportunistic or commensal and associated with disease only in individuals with impaired immune function. We have given the term ‘Opportunists’ to microbes in this class (Figure 1a).

Class 2: “Pathogens that cause damage either in hosts with weak immune responses or in the setting of normal immune responses”. In this category are microorganisms that cause host damage by both host- and pathogen-mediated mechanisms and are viewed as opportunists because their prevalence is higher in groups with impaired immune function. However, the capacity of Class 2 microorganisms to mediate disease in individuals with apparently normal immunity is indicative of the expression of microbial characteristics that promote their ability to evade normal host defenses that would otherwise eliminate them. We have termed these microbes ‘Equal Opportunists’ (Figure 1b).

Class 3: “Pathogens that cause damage in the setting of appropriate immune responses and produce damage at both ends of the continuum of immune responses”. In this category, microorganisms can cause damage in normal hosts, which is amplified in the setting of both weak and strong immune responses. We have termed these microbes ‘Bipolar Pathogens’ (Figure 1c).

Class 4: “Pathogens that cause damage primarily at the extremes of both weak and strong immune responses”. In normal hosts, microbes in this class cause relatively limited damage. However, a weak immune response can promote infection and microbe-mediated damage, while a strong immune response can produce excessive host-mediated damage. We have termed these microbes ‘Immunoreactive Opportunists’ (Figure 1d).

Class 5: “Pathogens that cause damage across the spectrum of immune responses, but damage can be enhanced by strong immune responses”. In this class, microorganisms cause infections that result in microbe-mediated damage but are associated with protracted or chronic damage resulting from an excessive or inappropriate immune response. We have termed these microbes ‘Immunoreactive Pathogens’ (Figure 1e).

Class 6: “Pathogens that can cause damage only in conditions of strong immune responses”. This class was first thought to be a largely theoretical category to describe a growing list of diseases that may have a microbial etiology not associated with impaired immune function. These organisms may also be members of the normal microbiota and confer a benefit to the host in settings of normal or weak responses. We have termed these microbes ‘Immunoreactive Commensals’ (Figure 1f).

## 3. *Candida* within the DRF

The ability of *C. albicans* to adapt to various and changing host environments (immune status, microbiota, anatomical location, etc.) is key to its ability to cause a wide range of diseases. Accordingly, *C. albicans* pathogenesis has often been described using phrases based on the concept that *C. albicans* is the exclusive causative agent that “converts from commensal to pathogen” or exists as an “opportunistic pathogen”. Under the tenets of the DRF, these phrases or terminology are no longer valid. In the following sections, we highlight the classification of various forms of candidiasis within the DRF and discuss strategies to reduce host damage, which may include immunomodulation and/or interventions that reduce or eliminate *C. albicans*. At mucosal surfaces, it is also conceivable that strategies to limit *C. albicans* host damage could result in commensalism, which could provide a host benefit. While this review/prospective article focuses specifically on *C. albicans*, we recognize that the DRF does not discriminate between individual *Candida* species. Thus, where appropriate we include information for other *Candida* species as well.

### 3.1. Class 1. Oropharyngeal Candidiasis: Damage Occurs Only in Situations of a Weakened or Compromised Immune System

Oropharyngeal candidiasis (OPC), commonly known as “thrush”, encompasses infections of the tongue and other oral mucous membranes, and may extend into the pharynx. OPC can present as white curd-like lesions (pseudomembranous) or reddened patches (erythematous). OPC is rare in healthy adults, occurring almost exclusively in immunocompromised individuals. In fact, OPC is the most common oral infection in the HIV^+^ population, although the incidence has been reduced significantly with antiretroviral therapies (ART) [8,9,10,11]. In addition to HIV infection, OPC occurs in 35% of cancer patients who have recently received chemotherapy, the elderly and infants, and those under conditions of malnutrition and local immune suppression (e.g., steroid inhalers for asthma). Further, patients with Sjogren’s syndrome, diabetes and other metabolic or hormonal disorders or those on antibiotics are also predisposed to OPC [8,9,12,13]. 

OPC is a biofilm-associated disease resulting from the adherence of yeast cells to mucosal surfaces followed by hyphal invasion, which is associated with secreted proteolytic enzyme expression [14]. Therefore, under conditions of weak immune responses, *C. albicans* invokes considerable damage upon the host, highlighting its Class 1 designation functioning as an ‘Opportunist’. However, as part of the microbiota in the oral cavity, *C. albicans* can exist asymptomatically in individuals with normal or strong protective host defenses. Under these conditions, *C. albicans* has the potential to develop a symbiotic relationship with the host, imparting a degree of protection against potentially harmful microbes that come in contact with the oral cavity [3]. This potential benefit in the context of strong host responses and damage in the face of weak responses defines a Class 1 pathogen.

Experimental and clinical evidence have provided significant advances in terms of understanding more specifically what contributes to host defense against OPC. While it is clear that innate defenses (salivary flow, antimicrobial peptides) help limit *C. albicans* overgrowth in the oral cavity, it became quite clear during the HIV epidemic that CD4^+^ T cells were the primary protective host defense mechanism against OPC [8,9,15,16,17,18,19,20]. An established mouse model of OPC [21] has been widely used for investigating *Candida* virulence factors, immune mechanisms against candidiasis and the efficacy of antifungal agents [4]. Originally, it was thought that the primary protective mechanism by CD4^+^ T cells was the Th1 phagocyte-dependent response [8]. However, the discovery of the Th17 axis and subsequent in vivo studies using the OPC model identified the CD4^+^ Th17 response as the primary protective response [22,23]. Importantly, in the absence of IL-17 and Th1/Th17-related cytokines IL-12 and IFN-γ, innate cell recruitment (predominantly neutrophils), activation, and phagocytosis of *C. albicans* cells fails to occur. Using knockout mice, defense against OPC was shown to be more dependent on Th17 than Th1-type immunity, with Th17-deficient mice exhibiting impaired neutrophil recruitment and high fungal burdens [24]. It is also noteworthy that Th17 cells enhance the expression of antimicrobial peptides including defensins and histatins, which are produced by the oral epithelial cells and salivary glands, respectively [25]. Therefore, the Th1/Th17 immune response is central to combating and preventing oral *Candida* infections in immunocompetent conditions, providing a large-scale benefit with little to no damage from a strong immune response. In addition to Th17 cells, there are also natural Th17 (nTh17) cells that have innate immune characteristics [26,27]. Unfortunately, there are no known phenotypic markers to distinguish Th17 cells from nTh17 cells. Moreover, based on mouse nTh17 cells that express CD4, it is speculated that if such cells exist in humans, they too would be depleted in HIV+ individuals and thus not available for protection against OPC. In contrast, although the incidence of OPC can be high in the absence of ART, it is postulated that under CD4^+^ Th1/Th17 immunocompromised conditions, secondary host defense mechanisms can provide some protection against infection. These include oral epithelial cells via annexin-A1 and CD8^+^ T cells, although the mechanisms are not well understood [8,28,29].

#### Strategies to Reduce Damage

**Figure 2:**
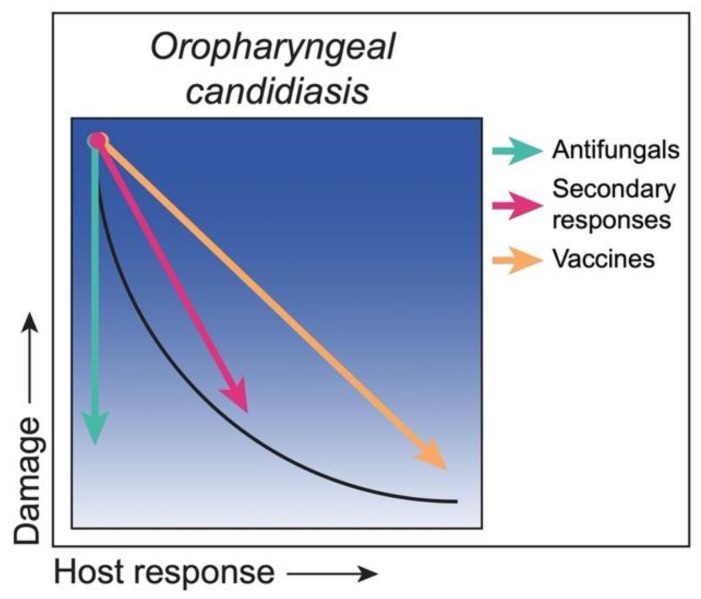
Class 1—Strategies to reduce damage. *C. albicans* as an ‘Opportunist’ during oropharyngeal candidiasis, with damage under weak responses and minimal to no damage under strong responses (black curve). Under conditions of weak immune responses, antifungal drugs (green arrow) reduce the fungal load, thereby reducing the associated host damage and disease-related symptoms. Boosting secondary host defense responses (pink arrow) reduces damage by bringing the host response to a near normal level. Therapeutic vaccines (orange arrow) would reduce damage by boosting the host response towards normal protective responses.

Under immunocompromised conditions, strategies to reduce damage can include actions against *C. albicans* and/or enhancing the immune response, resulting in a return to a state of asymptomatic colonization. Antifungal drugs are traditionally used to reduce the fungal burden and often result in the ablation of OPC. However, the most commonly used antifungal drugs for OPC are fungistatic (azoles), hence drug discovery strategies are focused on identifying fungicidal drugs. Areas of interest include small molecule libraries to identify drugs with antifungal activity and the repurposing of existing approved drugs that may have unbeknown antifungal activity. As alluded to above, secondary host defense mechanisms involving epithelial cells or CD8^+^ T cells may provide weaker but sustainable protection via CD4^+^ T cell-independent mechanisms [8,28,29]. Similarly, immunotherapies to enhance or achieve stronger Th17 responses would certainly enhance protection. Another active area of research is the development of vaccines that promote protective adaptive memory host responses to effectively target *C. albicans*. Currently, there are no approved vaccines for *C. albicans*, but there are several vaccine candidates in various stages of investigation, including clinical trials [30,31]. Although immunocompromised patients with inherited or acquired T cell deficiencies will not benefit from a T cell vaccine, it is possible that a B cell-antibody vaccine may be effective as long as the vaccine is given prior to the immunosuppression. If memory B cells and protective anti-*Candida* antibodies were generated, it would be possible to maintain some level of protective host response under acquired T cell immunosuppression. This may apply to HIV-infected people vaccinated prior to HIV exposure and associated immunosuppression. A summary of the strategies with their putative outcomes is illustrated in Figure 2.

### 3.2. Class 2. Hematogenously Disseminated Candidiasis (HDC): Damage Occurs in Hosts with Weak or Normal Immune Responses

Hematogenously disseminated candidiasis (HDC) arises when *Candida* spp. gain access to the bloodstream (candidemia), which can lead to deep-seated candidiasis (DSC; infection of internal organs), including intra-abdominal infection, peritonitis, and osteomyelitis [32]. *C. albicans,* along with other non-*albicans Candida* (NAC) species, were found to be the second most common cause of invasive infection in North American intensive care units (ICU), inclusive of all origins of infection in a 2009 study [33]. More recently, *Candida* spp. were found to be the seventh most frequently reported pathogen among all adult hospital-acquired infections in the United States, and the leading cause of central line-associated bloodstream infections in hospital wards and ICUs [34]. *Candida* spp. are currently ranked as the fourth leading cause of bloodstream infections in hospitalized patients and have been reported to cause up to 22% of hospital-acquired bloodstream infections in the US [35,36]. HDC is a leading cause of mycosis-associated mortality and once in the bloodstream, *C. albicans* can infect a wide range of target organs, including the kidney, spleen, liver, heart, gastrointestinal (GI) tract, and lungs [32]. Within target organs, invasive infections lead to significant tissue damage [37,38]. A major problem when managing patients with HDC is the difficulty in establishing the diagnosis. The sensitivity of blood cultures, the current diagnostic gold standard, is only ~50% across the spectrum of invasive candidiasis [39,40,41]. The poor performance of blood cultures, combined with their slow turn-around time, can lead to delays in the initiation of effective antifungal therapy, which contributes to the high mortality rates associated with *Candida* bloodstream infections [42]. 

HDC most commonly stems from the translocation of commensal *Candida* across damaged GI tract mucosa into the systemic circulation or direct inoculation from colonized vascular catheters. GI translocation is often seen in neutropenic and other immunocompromised hosts, in particular among cancer patients [43,44]. Central line catheter or other indwelling medical device-associated infections typically emerge from biofilms formed on these devices. Biofilms provide a niche for microorganisms where they are protected from both the host immune system and antifungals, and they serve as a reservoirs for cells with direct access to the bloodstream [45,46]. In most cases, the recommended therapy for these foreign body-associated infections is device removal [47]. If the device is not removed, continual seeding of the bloodstream can overwhelm intact host defenses in non-neutropenic patients with normal host responses, causing significant mortality despite antifungal therapy and intact immune functions [48,49,50]. Therefore, the weak defense in central line catheter-associated bloodstream infections is related to a breach in the barrier protecting systemic circulation, as opposed to a more specific immune deficiency. This damage is amplified in acutely ill patients with additional immune deficiencies who acquire HDC. Thus, the fact that HDC can occur under both weak and normal defenses, albeit disproportionately, demonstrates the ‘Equal Opportunist’ behavior of *C. albicans* in these circumstances and highlights its DRF Class 2 designation. The ability of *C. albicans* to cause systemic disease in immunocompetent individuals highlights the fact that the term “opportunistic” does not always apply to this organism. 

#### Strategies to Reduce Damage

As stated above, a major issue in treating patients with HDC is timely and effective diagnosis. Blood cultures are both ineffective (over half of patients with HDC are blood culture negative) and time-intensive (median time to positivity is 2–3 days) [39,40]. Therefore, therapeutic benefits that reduce damage could be achieved through the development of novel and rapid diagnostic tools that would allow for antifungal therapies to be administered at earlier timepoints during infection. Two non-culture diagnostics have been approved by the US Food and Drug Administration (FDA) for clinical use, the serum β-D-glucan assay (BDG; Fungitell assay from the Associates of Cape Cod available in the US) and the T2Candida nanodiagnostic panel (T2 Biosystems, Lexington, MA). The BDG assay detects any fungal (1,3)-β-D-glucan in serum samples and is therefore not specific for candidemia. By contrast, the T2Candida panel can differentiate the five most common *Candida* species in approximately five hours, however, the panel requires a dedicated instrument [51,52]. In addition to the BDG and T2Candida assays, several other non-culture diagnostic techniques have been developed, including mannan/anti-mannan antibody assays [53], *C. albicans* germ tube antibodies (CAGTA), and various PCR-based assays. However, while the mannan/anti-mannan assays and CAGTA tests are used in Europe, they have not gained FDA approval in the US. Furthermore, the utility of these non-culture-based tests depends on a number of clinical factors that have yet to be sufficiently investigated. 

**Figure 3:**
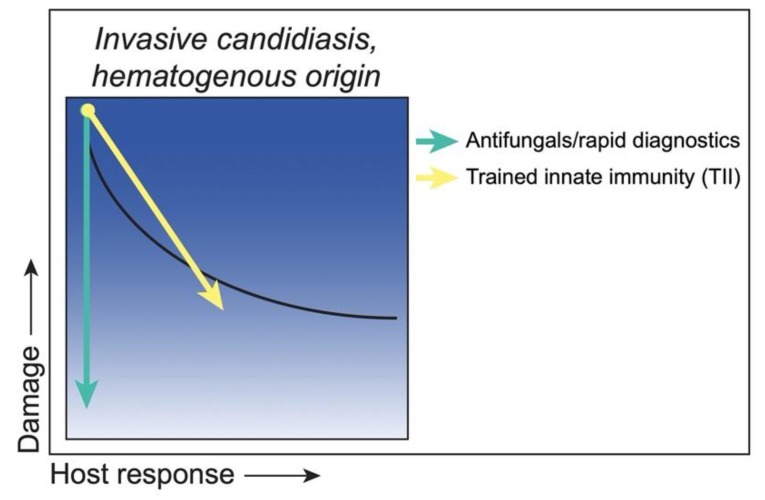
Class 2—Strategies to reduce damage. *C. albicans* as an ‘Equal Opportunist’ during invasive candidiasis of hematogenous origin, with damage under weak responses and moderate damage remaining under strong responses (black curve). Antifungals (green arrow), or rapid diagnostics leading to more timely antifungal administration, reduce the fungal load, thereby reducing host damage together with symptoms of disease. Of note, some antifungals (i.e., amphotericin B) can also have immunomodulatory properties, which would result in a shift toward a greater host response in some cases. Trained innate immune (TII) strategies (yellow arrow) would boost the innate host response, resulting in reduced fungal load and a reduction in the associated host damage to moderate levels similar to that seen with normal host responses.

In addition to the traditional vaccine strategies that induce protective adaptive responses (memory T and/or B cells), another area of interest is trained innate immunity (TII), which refers to a non-specific memory immunity mediated by innate cells that results in an enhanced immune response to secondary challenge [54,55]. Using a murine intravenous model, Quintin et al. demonstrated that *C. albicans* and fungal β-glucan can induce epigenetic reprogramming of monocytes that results in increased cytokine production and protection against secondary lethal challenge that is T and B cell-independent [56]. Subsequent studies have shown that this epigenetic reprogramming occurs at the level of hematopoietic stem and progenitor cells (HSPCs), which are expanded in response to training and undergo metabolic reprogramming [57,58]. Lilly et al. demonstrated that intraperitoneal inoculation with *C. dubliniensis* could reach the murine bone marrow compartment and induce a conceptually similar trained innate protection against intravenous *C. albicans* infection [59]. Similar training of HSPCs and monocytes has been shown following Bacillus Calmette–Guérin (BCG) vaccination [60,61]. This training is non-specific, as BCG vaccination, β-glucan exposure, and other TII stimuli have been shown to provide cross-protection against several bacterial and fungal pathogens [62]. Trained innate immune protection is of particular interest when considering immunosuppressed populations who may not respond to traditional vaccination strategies. Furthermore, the apparent role of trained monocytes in the protection against systemic candidiasis is intriguing when considering neutropenic populations. However, the importance of trained innate immune protection still needs to be verified clinically. A summary of these strategies and putative outcomes is illustrated in Figure 3.

### 3.3. Class 3. Intra-Abdominal Candidiasis: Damage Occurs throughout the Continuum of Immune Responses but Is Amplified at Extremes of Both Weak and Strong Immune Responses

Intra-abdominal candidiasis (IAC) results from the entry of *C. albicans* into the abdominal cavity through translocation of the GI mucosal barrier, or by direct inoculation, which can occur via contaminated peritoneal dialysis catheters or as a result of the perforation of the GI tract [63,64,65,66]. While HDC is a Class 2 disease, IAC is considered a Class 3 disease. Even in otherwise immunocompetent individuals, inoculation of the abdominal cavity can cause peritonitis, an inflammatory disease of the lining of the abdominal cavity, which can be amplified by an uncontrolled host response, leading to host-mediated damage. The cardinal clinical signs and symptoms of peritonitis include fevers, chills and abdominal pain, and complications of peritonitis include invasion of adjacent organs, such as the liver and spleen, and/or abscess formation [67,68]. If untreated, intra-abdominal candidiasis can disseminate via the bloodstream (HDC) and cause DSC. In fact, it is estimated that secondary candidemia occurs in 5%–20% of IAC cases [69]. This may be exacerbated in immunocompromised patients that lack innate defenses that could help to contain or control *C. albicans* within the peritoneal cavity. Using a casein peritonitis model, it was recently shown that *C. albicans* induced rapid recruitment of peritoneal Ly6G^+^ neutrophils and triggered neutrophil extracellular trap formation (NETosis) [70]. This was dependent on dectin-2 signaling and was required to restrain the spread of *C. albicans* from the peritoneal cavity to the kidneys, exemplifying the need for innate defenses in controlling peritoneal infection. IAC is distinct from HDC (Class 2), in that host damage is equally severe during a strong response, due to the local nature of the peritoneal immune response, which is characterized by neutrophil recruitment and inflammatory cytokine production [69]. While defects in innate defenses are associated with susceptibility to IAC, the robust response could ultimately compound the outcome of infection and lead to peritonitis and sepsis [71]. The severity of these infections at either end of the host response continuum, with some damage occurring even under optimal host responsiveness, leads to *C. albicans* being classified in this scenario as Class 3 with the behavior as a ‘Bipolar Pathogen’.

#### Strategies to Reduce Damage

Treatment for IAC usually entails both source control with drainage and surgical intervention and prompt antifungal therapy. Echinocandin antifungal drugs are often used as first-line agents, but clinical efficacy is often variable. This variability may be due in part to limited penetration within intra-abdominal lesions [72]. However, another unique feature of intra-abdominal infections (IAI) involving *C. albicans* is that they are often polymicrobial, and fungal-bacterial infections are associated with higher mortality rates than polymicrobial bacterial infections [73,74,75,76]. A murine model of polymicrobial IAI involving co-inoculation of *C. albicans* with the bacterial pathogen *Staphylococcus aureus*, showed similar synergistic effects on mortality compared with mono-microbial inoculation that included the dissemination of both species and subsequent sepsis [77]. A significant reduction in mortality was achieved by prophylactically targeting PGE_2_ production with a non-steroidal anti-inflammatory drug (NSAID) [78]. Although these data demonstrate that NSAID treatment exerts a protective effect during polymicrobial IAI, global inhibition of both COX-1 and COX-2 pathways may be non-therapeutic and/or detrimental following infection clinically. There are several murine and human studies that have examined the effects of non-selective or COX-2 selective eicosanoid inhibitors during peritonitis or sepsis. Some studies supported a protective effect, while others, particularly in humans, were inconclusive [79,80,81,82]. However, it is likely that COX-1 is responsible for the initial production of PGE_2_ because resident peritoneal macrophages are pre-loaded with this enzyme, while COX-2 is transcribed/translated only after cells are stimulated. Further evidence from the experimental model supports targeting COX-1, along with PGE_2_ receptors EP1 and EP3 as possible therapeutic strategies to restrain lethal inflammation during IAC along with traditional antifungal drugs [83].

**Figure 4:**
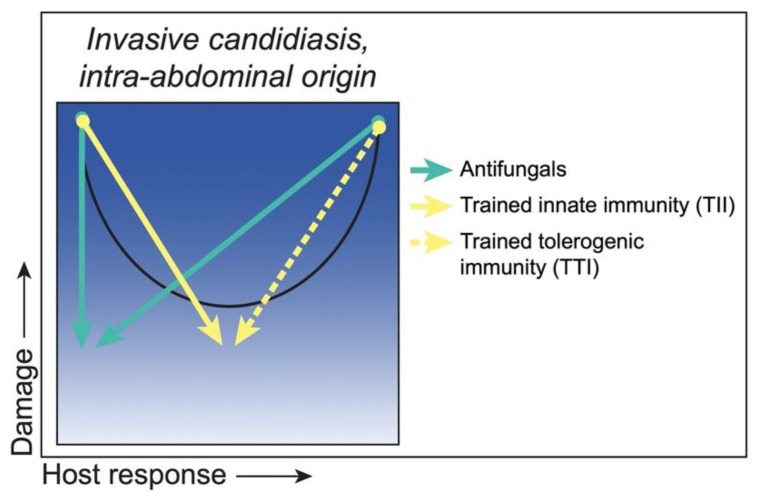
Class 3—Strategies to reduce damage. *C. albicans* as a ‘Bipolar Pathogen’ during invasive candidiasis of intra-abdominal origin, with damage under strong (hyper) and weak (hypo) responses and moderate damage under moderate responses (black curve). Antifungals (green arrows) reduce fungal load, thereby reducing both fungal-associated host damage under conditions of low host responses and host damage caused by hyperimmune reactivity toward the organism. Trained innate immunity (TII) (yellow arrow) would boost hypoimmune responses into the normal host response level, while trained tolerogenic immunity (TTI) (dashed yellow arrow) would suppress hyperimmune responses back to the normal level concomitant with minimal host damage.

There is also the possibility of inducing protective trained innate responses during IAC. In the same polymicrobial IAI model mentioned above, it was demonstrated that low virulence/low damage *Candida* species induce long lived protection mediated by innate cells [59,84]. This protection is thought to be driven by a novel form of trained innate immunity mediated by Gr-1^+^ myeloid-derived suppressor cells (MDSCs) called “trained tolerogenic immunity” (TTI) [85]. MDSC accumulation has been described in patients with sepsis, as well as in in vivo sepsis models. Whether the mechanism of protection conferred by trained MDSCs includes robust antifungal defenses, similar to trained monocytes, or is limited to direct suppression of the sepsis proinflammatory response remains to be determined. On the other hand, cases of IAC dissemination with concomitant immunosuppression may benefit from TII strategies to boost innate defenses similar to those described for HDC. A summary of these proposed strategies and putative outcomes is illustrated in Figure 4.

### 3.4. Class 4. Gastro-Intestinal Candidiasis: Damage Occurs Primarily at the Extremes of Both Weak and Strong Immune Responses

*C. albicans* is considered a natural inhabitant of the human GI tract and can be maintained at various levels without overt host damage. Environmental factors such as antibiotic use and diet, which can alter bacterial microbiota levels and lower colonization resistance, lead to the overgrowth of *C. albicans* in the GI tract in humans [86,87]. Overgrowth may facilitate localized mucosal infection and/or generalized GI tract disturbances, but the validity of GI candidiasis via overgrowth as a clinical entity is controversial [88,89]. This has been difficult to verify due to the lack of specific symptoms (belching, bloating, indigestion, nausea, diarrhea, and gas) and lack of diagnostic tests. Patients considered to be at risk for this type of candidiasis include cancer and transplant patients receiving immunosuppressive therapy or prolonged antibiotic prophylaxis, and therefore, it would represent a disease associated with impaired host immunity [90,91]. Experimental murine models have indicated a role for both innate and adaptive immunity (particularly T cells) in controlling GI candidiasis [92,93,94,95,96,97]. At the other end of the host response spectrum, experimental and clinical evidence also suggests that *C. albicans* GI colonization may be a cofactor for inflammatory diseases, which could be considered host-mediated damage. Therefore, based on current, albeit limited data, GI candidiasis fits within the Class 4 designation, with *C. albicans* behaving in this case as an ‘Immunoreactive Opportunist’, causing host damage in the context of both strong and weak host responses.

#### Strategies to Reduce Damage

**Figure 5:**
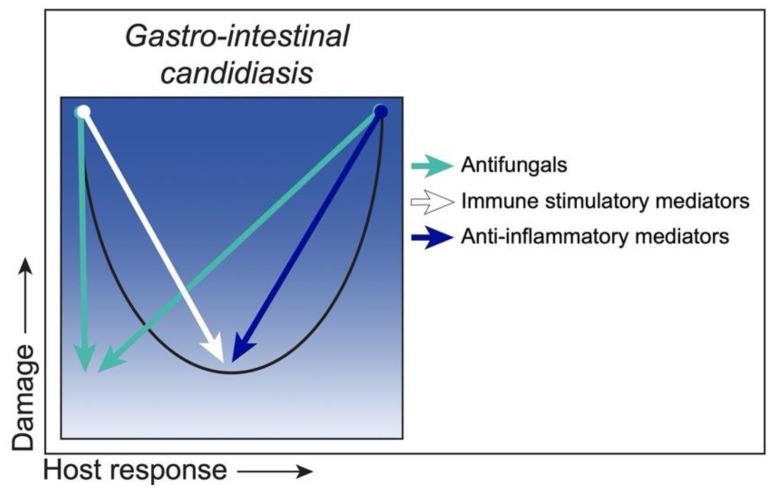
Class 4—Strategies to reduce damage. *C. albicans* as an ‘Immunoreactive Opportunist’ in gastro-intestinal candidiasis, with mucosal damage occurring under weak (hypo) or strong (hyper) host responses and minimal damage under moderate responses (black curve). Antifungals and/or probiotics (green arrows) reduce fungal loads and promote healthy levels of GI colonization, thereby reducing host damage associated with *Candida* overgrowth or hyperimmune activation. Immune stimulatory mediators would boost hypoimmune responses (white arrow), while anti-inflammatory mediators (blue arrow) would suppress hyperimmune responses, both resulting in a return to the homeostatic host response level and minimal host damage.

It is clear from various animal models that the major defense against *C. albicans* overgrowth in the GI tract is the presence of the bacterial microbiota and normal gut peristalsis. Antibiotic treatment or the use of germ-free gnotobiotic animals promotes consistent *C. albicans* colonization of the gut [98]. In particular, antibiotics that deplete anaerobes facilitate the highest *C. albicans* GI colonization levels [99], supporting the concept that a balanced GI microbiota is important in preventing the overgrowth of potential pathogens (pathobionts) [100]. Mechanistically, anaerobic bacteria induce expression of HIF-1α (a key regulator of innate immunity), leading to increased expression of LL-37 antimicrobial protein by intestinal epithelial cells [99]. Pharmacologic activation of colonic HIF-1α resulted in a significant reduction of *C. albicans* GI colonization and decreased dissemination and mortality, supporting the concept that boosting innate defenses is beneficial against GI candidiasis. This also indicates that strategies to promote a balanced microbiota with increased anaerobic bacteria may also lead to protection against fungal overgrowth. However, a clinically proven probiotic and/or diet-based therapy has not yet been identified and efficacy would likely be dependent on multiple clinical variables. As also alluded to, strategies that boost or subdue innate responses, immune stimulatory mediators or anti-inflammatory mediators, respectively, will also be beneficial. A summary of these strategies and putative outcomes is illustrated in Figure 5.

### 3.5. Class 5. Denture Stomatitis: Damage Occurs across the Spectrum of Immune Responses, but Damage Is Enhanced by Strong Immune Responses

*Candida-*associated denture stomatitis (DS) is by far the most common form of oral candidiasis, with prevalence rates as high as 70% among partial or complete denture wearers [101,102,103,104]. DS is a chronic inflammatory condition manifested in the oral mucosa in direct contact with the dentures, typically as mild to severe forms of palatal edema and erythema, papillary hyperplasia (small pebble-like sores) and petechial hemorrhage (pinpoint bleeding) [102,105,106,107]. *C. albicans* in the oral cavity readily adheres to the acrylic denture materials and forms biofilms, resulting in the continuous seeding of the biofilm-associated pathogens onto the palate mucosa [103,108,109,110]. Thus, biofilm formation is believed to be the key pathogenic process of DS that leads to chronic infection of the affected tissues. Infection is particularly exacerbated in situations involving ill-fitted dentures causing frictional irritation and damaging the mucosal barrier, which in turn could facilitate fungal tissue invasion [13]. Under such conditions, a strong immune response is continuously triggered and invokes host-mediated damage, whereas a weak to moderate immune response minimizes damage. Therefore, DS is representative of DRF Class 5 with *C. albicans* acting as an ‘Immunoreactive Pathogen’. In support of this, transcriptome analyses of palatal tissues of *C. albicans*-positive DS subjects showed strong upregulation of signaling pathways involving neutrophil recruitment, toll-like receptor signaling and T cell activation, compared to their healthy counterparts [111].

In addition to *C. albicans*, cases of symptomatic DS involving NAC species have been increasingly reported. Among patients diagnosed with *Candida*-associated DS, NAC species are detected in approximately 30%–40% of patients, most commonly *C. glabrata, C. tropicalis* or *C. krusei* [112,113,114,115,116,117,118,119]. Recent studies indicated that the severity of DS was associated with the presence of mixed-*Candida* species, particularly *C. tropicalis* with the highest degree of palatal inflammation [117,118].

A rat model of DS using a custom-fitted removable denture system has been developed and exploited to further understand a role of *C. albicans* biofilms in DS pathogenesis in vivo [120]. Longitudinal analyses of inoculated rats demonstrated sustained *C. albicans* colonization and biofilm formation on both denture and palate tissue and resulted in marked inflammation in the palate mucosa similar to clinical DS [121]. Subsequent studies using *C. albicans* mutants defective in morphogenesis (*efg1*) and biofilm formation (*bcr1*) revealed that inoculated denture-bearing rats had minimal tissue damage and normal weight gain in the absence of mature biofilms, compared to those inoculated with robust biofilm-forming strains [122]. These results provided the first direct evidence to support the clinical hypotheses and a pivotal role for these central regulators of *C. albicans* biofilm formation and virulence in mucosal tissue damage during DS. In addition to *C. albicans*, a recent study evaluated the virulence and pathogenicity of *C. glabrata* in the rat DS model. While *C. glabrata* readily established colonization on denture and palate, it had no apparent role in inducing DS pathology nor could it enhance *C. albicans* pathogenicity under co-inoculated conditions [123]. How other clinically relevant *Candida* species (*C. tropicalis* and *C. krusei*) exhibit virulence in the rat model has yet to be determined.

#### Strategies to Reduce Damage

**Figure 6:**
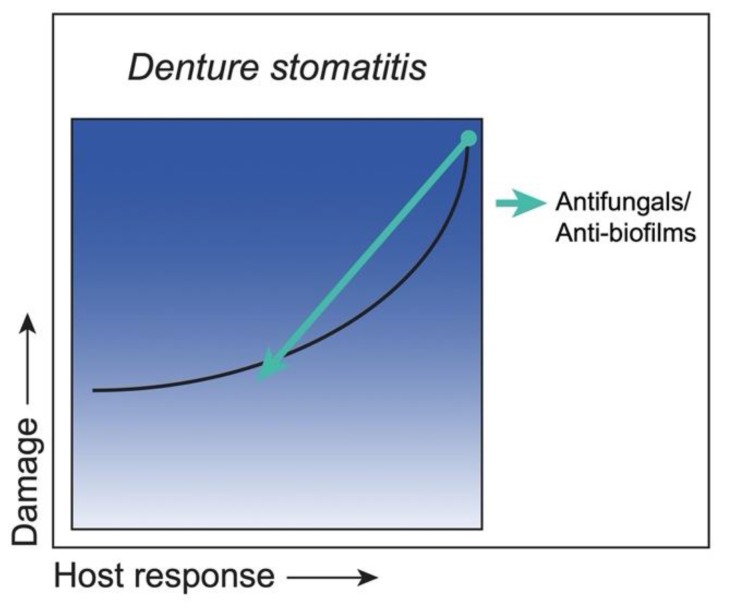
Class 5—Strategies to reduce damage. *C. albicans* as an ‘Immunoreactive Pathogen’ in denture stomatitis, with mucosal damage occurring across a spectrum of host responses (black curve). Removal of biofilms from dentures and clearance of palatal colonization reduce immune responses, thereby diminishing host damage. Strategies include antifungal therapies via denture-optimized delivery systems and the use of anti-biofilm agents that inhibit fungal adherence to denture materials (green arrow), all of which would minimize both host and biofilm-mediated damage.

Considering abundant evidence indicating that the severity of clinical DS is directly associated with the damage caused by *C. albicans* biofilms, there is a demand for better biofilm control strategies that inactivate and remove existing biofilms, as well as to prevent future regrowth on denture materials. Although antifungal therapy is effective for the treatment of acute infections, the effect is only short-term, with high recurrence rates [124,125,126,127]. A recent strategy developed for enhanced drug delivery and new drug discovery involves modified acrylic denture base resins containing microbicide-conjugated and releasing methacrylate monomers (DABCO). These denture base resins have been shown to exert exceptional fungicidal activity, with minimal cytotoxicity in vitro [128]. Current studies are testing the resins in the rat DS model. In addition to the custom-fitted denture model system, a three-dimensional (3D) digital fabrication of a universal-fitting rodent denture system has recently been reported [129]. This 3D fabrication method includes a modified design optimized for delivery of topical drugs onto the rat palate for short-term therapeutic evaluations. Another strategy to overcome fungal overgrowth during DS is to inhibit adherence of the organisms on denture base materials by countering the hydrophobic properties of acrylic resins that promote *C. albicans* adhesion and colonization [130,131]. Accordingly, hydrophilic coating materials [132] and polysaccharides, namely mannans and chitosan [133,134,135], have been shown to inhibit fungal adhesion to acrylic denture materials. Yet another anti-biofilm strategy being explored is photodynamic inactivation (PDI) therapy. Several studies have indicated that PDI has anti-*Candida* activity equivalent or superior to nystatin or azole drugs and resulted in reduced palatal inflammation in patients with DS [136,137,138]. These strategies/outcomes are summarized cumulatively in Figure 6.

### 3.6. Class 6. Vulvovaginal Candidiasis: Damage Occurs Only under Conditions of Strong Immune Responses

Similar to the oral cavity and GI tract, *C. albicans* is present as part of the normal vaginal microbiota, but overgrowth can initiate infection and cause vulvovaginal candidiasis (VVC). VVC is characterized by itching, burning and redness of the vulva and vaginal mucosa, often accompanied by white vaginal discharge [139,140,141,142,143,144]. With high prevalence worldwide, an estimated 75% of otherwise healthy women of childbearing age are affected by VVC at least once in their lifetime, with an additional 5%–8% suffering from chronic or recurrent VVC (RVVC) [139,143,144]. Various predisposing factors have been reported to trigger acute VVC (e.g., high-estrogen oral contraceptive use, hormone replacement therapy, antibiotic usage, uncontrolled diabetes mellitus), while onset of RVVC is considered idiopathic [139,145]. Clinically, evidence indicates that robust migration of polymorphonuclear neutrophils (PMNs) into the vaginal cavity is a key pathogenic process in symptomatic VVC/RVVC, leading to chronic tissue damage concomitant with persistent fungal overgrowth [142]. This is in the absence of any role or function of adaptive immunity [146,147,148,149,150,151,152,153,154] in direct contrast to the oral cavity. Conversely, asymptomatic colonization in the absence of an aggressive PMN response is representative of the commensal relationship, irrespective of vaginal fungal burden. Therefore, VVC/RVVC represents DRF Class 6, in that host-mediated damage occurs only under a strong immunoreactive state to *C. albicans,* which otherwise persists innocuously as a commensal (‘Immunoreactive Commensal’).

An estrogen-dependent mouse model of VVC closely parallels the human disease and has advanced the knowledge on VVC pathogenesis that is translatable to women [155]. Similar to humans, robust PMN migration occurs following vaginal inoculation in mice and represents the hallmark of VVC-associated host damage [155,156]. However, it is important to note that there is evidence of vaginal tissue damage in inoculated mice lacking PMNs (i.e., absence of a robust host response) [157]. As such, VVC could potentially be assigned to DRF Class 5 with detectable host damage in the absence of a host response. Another interesting caveat is that unlike the protective role for Th17-type responses against OPC detailed in DRF Class 1, the PMN response during VVC occurs independent of the Th17 pathway, with any detectable IL-17 deemed dispensable [158,159]. 

It is postulated that the immunopathogenic PMN response is triggered by the sensitivity of the vaginal epithelium to *C. albicans* [142,156] and largely dependent on a threshold level of *C. albicans* burden that defines a susceptible vs. resistant phenotype [156]. During an immunopathologic response, several inflammatory effectors, namely S100A8 and IL-1β secreted by epithelial cells and further by migratory PMNs, are considered key inflammatory markers of VVC immunopathology [160,161]. Under resistant conditions, the epithelial cells instead hold *C. albicans* growth in check in a non-inflammatory manner mediated by annexin-A1 [162,163,164], thus limiting unnecessary host damage and promoting the commensal relationship. 

Several virulence properties of *C. albicans* have been investigated for a pathogenic role in vaginitis. Clinical and experimental studies have shown pathogenic roles for *C. albicans* hyphal formation, hypha-associated adhesion molecules, host recognition receptors, and select secretory aspartyl proteinases (SAPs) [157,165,166,167,168,169]. More recently, Candidalysin, a newly discovered hypha-derived toxin, has been shown to be a major virulence determinant for vaginitis [161]. Finally, a pathogenic role for *C. albicans* biofilm growth on vaginal epithelium has been suggested from animal studies [28], although the clinical significance of biofilms in VVC remains unknown [170]. In direct contrast to *C. albicans*, *C. glabrata*, the second most common cause of VVC/RVVC [171,172,173], lacks morphological switching and hypha-related virulence traits. Despite this, *C. glabrata* has been suggested to have a considerable impact on *C. albicans* pathogenicity in other candidiasis models [174,175]. However, data from the mouse VVC model failed to suggest any immunopathogenic role for *C. glabrata* with or without *C. albicans* [176,177].

The hallmark of this DRF Class 6 scenario is a strong immunopathogenic condition locally in the vagina, characterized by strong host-mediated acute inflammation. This is highlighted further by the lack of any reduction in vaginal fungal burden, despite the strong migration and presence of activated PMNs, that ultimately leads to chronic mucosal damage if not treated. A recent study provided a potential mechanism for this unique PMN dysfunction in the vaginal environment. In mice that maintain consistent levels of *C. albicans* following inoculation, vaginal-associated heparan sulfate (HS) was identified as a competitive ligand for a key receptor interaction between PMNs and *C. albicans* that inhibited antifungal activity [160]. In contrast, a lone mouse strain resistant to symptomatic VVC (CD1) lacked the HS inhibitory effect concomitant with timely fungal clearance and resolution of inflammation. This inhibitory action by HS on PMN antifungal activity that predisposes susceptible mice to symptomatic VVC has been termed ‘neutrophil anergy’ [178]. Clinical studies are underway to assess this putative mechanism in women. 

An interesting question is whether oral thrush (OPC), that can occur following antibiotic usage, is at all similar to VVC as a DRF Class 6 scenario under those circumstances. This is unlikely for the following reasons: Although thrush following antibiotic usage may occur in otherwise healthy people, it is most common in children and older adults, whose immune systems are not yet fully developed or have declined, respectively. Under such conditions, it is likely that the overgrowth of *Candida* from the antibiotics and resulting biofilm formation overwhelms the host response(s) available. This is quite different to VVC where the overgrowth of *Candida* triggers a host response that is ultimately dysfunctional leading to the immunopathogenic condition. Both infections, however, require similar treatment via antifungals. It is quite possible that oral thrush as a result of antibiotic usage may be more akin to the DRF Class 5 scenario (denture stomatitis) where the host- and drug-resistant tissue-associated biofilm continues to stimulate chronic-type host responses that cannot reduce the fungal burden back to commensal status/homeostasis.

#### Strategies to Reduce Damage

**Figure 7:**
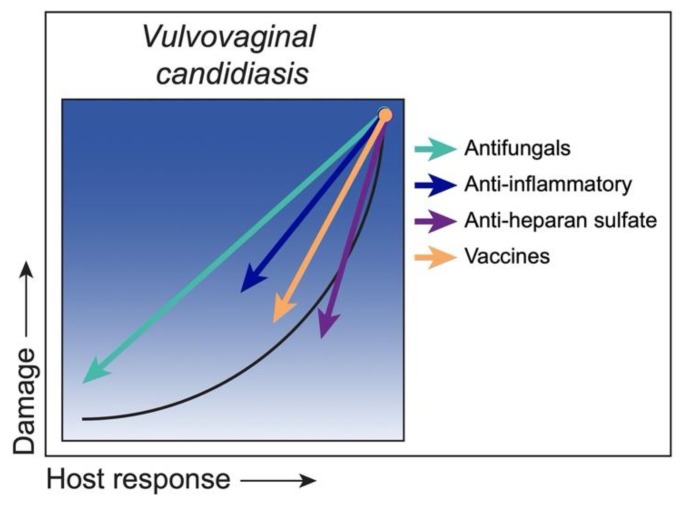
Class 6—Strategies to reduce damage. *C. albicans* as an ‘Immunoreactive Commensal’ in vulvovaginal candidiasis, with damage occurring from a hyper/aggressive inflammatory response by PMNs versus commensalism, characterized by a weak host response with little to no damage (black curve). Antifungal therapies temporarily eliminate *C. albicans* burden, thereby diminishing immune responses and related damage to an asymptomatic state (green arrow). Alternatively, anti-inflammatory therapies reduce damage by modulating the PMN response, while *C. albicans* colonization remains, albeit rather asymptomatically (blue arrow). Conversely, strategies to target heparan sulfate to enhance PMN antifungal activity during an immunoreactive state will promote *C. albicans* clearance, followed by timely resolution of inflammation-associated damage (purple arrow). Finally, a vaccine strategy would reduce damage by either eliminating/reducing *C. albicans*, or possibly through immunomodulation (orange arrow).

Antifungal drugs have been at the forefront of eliminating *C. albicans* or reducing it to commensal levels. Variable results, however, with existing fungistatic antifungal drugs and emergence of resistance have necessitated the search for new and improved drug regimens. Notably, the positive action by antifungal drugs will also attenuate the organism-mediated trigger of the immunopathogenic response. In addition to drug regimens, certain epitope-specific antibodies characterized as ‘protective’ antibodies have been used therapeutically against infection in mice [179,180,181,182] with a similar outcome. In either case, effective strategies to reduce the organism burden should, at least temporarily, result in a full return to an asymptomatic state under a homeostatic weak host response. Another strategy is to modulate the proinflammatory response by reducing/blocking/inhibiting inflammatory mediators or attenuating vaginal PMN migration. In such cases, *C. albicans* may remain at a considerable level, but its presence is largely asymptomatic with the host response subdued. While not evaluated fully for immune mediators or PMN presence, an open-label study testing zafirlukast, a leukotriene receptor antagonist for treatment of asthma, reported symptom-free conditions in RVVC patients after a long-term daily regimen [183]. As a clear example of drug repurposing, these immunomodulatory approaches offer substantial advantages with firm evidence for safety and effectiveness. Other approaches include probiotics (e.g., *Lactobacillus* spp.), or synthetic or natural-based compounds (e.g., polyphenols) that have been shown to have anti-inflammatory effects [184,185,186,187,188,189]. On the other hand, strategies to restore PMN antifungal activity during vaginitis by targeting HS would conceivably reduce the organism burden with a functional PMN response and ultimately restore the asymptomatic state. Although counterintuitive, based on the immunopathogenic nature of VVC and the lack of any role for adaptive immunity in protection against infection, a therapeutic vaccine approach for RVVC patients is another strategy that is currently under investigation. Accordingly, a recent phase II clinical trial using the Als3p vaccine (NDV-3A; NovaDigm Therapeutics) in RVVC patients showed significant efficacy in reducing symptoms [31] and clear evidence of induced antibodies [190] that are protective in mice [191]. Although the mechanism of action in humans remains unclear, it is also possible that the vaccine induces a concomitant or exclusive immunoregulatory effect, as postulated by Casadevall and Pirofski [182]. In contrast with the TII-induced protection against lethal *Candida/Staphylococcus* IAI/sepsis in mice, the *C. dubliniensis*-induced TII failed to mediate protection against experimental VVC [59]. The described strategies and putative outcomes are depicted in Figure 7.

## 4. Concluding Remarks

We have taken the opportunity to revisit the DRF in the context of *Candida* infections, with their unique categorizations into one of the six classes, depending on the site of infection and overall host response/pathogenesis properties [7]. Accordingly, we provide updates where available regarding the class-specific categorization for each type of infection. It is interesting to refer to the various DRF curves in terms of the type of pathogenesis. Class 1 (OPC) and Class 2 (invasive candidiasis of hematogenous origin) are clearly reflective of typical ‘pathogenesis’, with the majority of damage occurring in the absence of strong host responses. In contrast, Class 5 (DS) and Class 6 (VVC) are more appropriately reflective of ‘immunopathogenesis’, with the majority of damage occurring under strong host responses. Class 3 (invasive candidiasis of intra-abdominal origin) and Class 4 (GI candidiasis) are hybrids of pathogenesis and immunopathogenesis, depending on the circumstances surrounding the host response and resulting damage. These hybrid conditions are clearly more complicated in both diagnosis and treatment, with multiple means for damage to occur. However, the shear ability to recognize the possible infection scenarios in terms of the DRF enables a fuller understanding of the condition, which can aid decisions to optimize treatment and care.

While this review focuses specifically on *Candida* infections, and primarily *C. albicans*, the opportunity to apply the exercise of classifying fungal pathogenesis to the DRF can be done for any medically important fungal infection/disease and is strongly encouraged. Similar to *C. albicans*, *Aspergillus* spp. can cause a variety of diseases whose outcomes are largely dependent on the host response [192], therefore, applying the DRF framework to this organism could be very informative. While the majority of fungal pathogens will not fit multiple DRF classes, it will be interesting to compare and contrast the various fungal infections relative to the DRF and the anatomical site-specific tropisms. Furthermore, it is anticipated that similar strategies to reduce damage may apply as well to other fungal pathogens. As an example, Pirofski and Casadevall [193] recently discussed the classification of *Cryptococcus neoformans* as an organism that can cause damage in the setting of weak or strong immune responses (Class 3 or 4) and provided a similar commentary on proposed therapeutic strategies depending on the host response. 

Related to treatment and care, we have offered a number of strategies to reduce host damage in light of the DRF class-specific scenarios with *C. albicans*. Accordingly, some strategies target the organism independent of the host response, whereas others target the host response independently of the organism. These strategies are rarely mutually exclusive, as targeting the organism or the host independently will often have indirect effects on the other. Irrespective, the goal is to limit host damage below the disease threshold. We recognize that many of the class-specific strategies offered are speculative, based largely on data from animal models, and ultimately dependent on the level of damage (position along any one DRF curve). Fungal burden will also have an influence on any treatment strategy and will likely vary tremendously depending on the infection/site. It is difficult to assign a range of fungal burden in any one DRF class pathogenesis, due to many factors. This will also be quite different for animal models versus humans. Suffice to say, that at least for humans, the fungal burden during mucosal disease will be higher than commensal levels. Other factors will include the level of local or systemic host response, the site of infection, and the robustness of the pathogen. Nevertheless, our intent is to provoke thought into treatment strategies in light of the condition/scenario, that will ultimately change the positioning on the DRF curve to that which approaches homeostasis and ultimate recovery for the patient. 

## Abbreviations

**ART**	Antiretroviral therapy
**BCG**	Bacillus Calmette–Guérin
**CAGTA**	*C. albicans* germ tube antibodies
**DABCO**	1,4-Diazabicyclo[2.2.2]octane
**DRF**	Damage response framework
**DS**	Denture stomatitis
**DSC**	Deep-seated candidiasis
**GI**	Gastrointestinal
**HDC**	Hematogenously disseminated candidiasis
**HIV**	Human immunodeficiency virus
**HS**	Heparan sulfate
**HSPC**	Hematopoietic stem and progenitor cell
**IAC**	Intra-abdominal candidiasis
**IAI**	Intra-abdominal infection
**ICU**	Intensive care unit
**IFN**	Interferon
**IL**	Interleukin
**MDSC**	Myeloid-derived suppressor cell
**NAC**	Non-*albicans Candida*
**NSAID**	Non-steroidal anti-inflammatory drug
**nTh17**	Natural T helper type 17
**OPC**	Oropharyngeal candidiasis
**PDI**	Photodynamic inactivation
**PMN**	Polymorphonuclear neutrophil
**RVVC**	Recurrent vulvovaginal candidiasis
**SAP**	Secretory aspartyl proteinase
**Th**	T helper
**TII**	Trained innate immunity
**TTI**	Trained tolerogenic immunity
**VVC**	Vulvovaginal candidiasis
